# Assessment of Prognostic Value of Aspartate Aminotransferase-to-Platelet Ratio Index in Patients With Hepatocellular Carcinoma: Meta-Analysis of 28 Cohort Studies

**DOI:** 10.3389/fmed.2021.756210

**Published:** 2021-11-26

**Authors:** XinYue Zhang, Zhen Svn, MengSi Liv, MengNan Liu, YiHan Zhang, Qin Sun

**Affiliations:** ^1^Affiliated Hospital of Traditional Chinese Medicine, School of Integrated Traditional Chinese and Western Medicine, Southwest Medical University, Luzhou, China; ^2^Hengyang Medical School, University of South China, Hengyang, China; ^3^Department of Cardiovascular Medicine, National Traditional Chinese Medicine Clinical Research Base, Hospital Affiliated to Southwest Medical University, Luzhou, China; ^4^Medical Record Room, Affiliated Hospital of Traditional Chinese Medicine, Southwest Medical University, Luzhou, China; ^5^National Traditional Chinese Medicine Clinical Research Base, Drug Research Center of Integrated Traditional Chinese and Western Medicine, Affiliated Traditional Chinese Medicine Hospital, Southwest Medical University, Luzhou, China

**Keywords:** hepatocellular carcinoma, aspartate aminotransferase-to-platelet ratio index, APRI, noninvasive biomarker, prognosis, overall survival, disease-free survival, meta-analaysis

## Abstract

**Background:** Hepatocellular carcinoma (HCC) is one of the most common malignant tumors globally; it is valuable to predict its prognosis after treatment. Aspartate aminotransferase-to-platelet index (APRI), a non-invasive biomarker consists of two routine test parameters easily available in all the patients. Our study aimed to investigate whether APRI can serve as an independent prognostic marker in the patients with HCC.

**Methods:** We extensively searched PubMed, Embase, and Web of Science databases on June 20, 2021 to determine all relevant literature. The studies that explored the association between the APRI levels and prognosis of patients with HCC and reported risk estimate data were included. The Newcastle-Ottawa Scale was used to assess the quality of the included studies.

**Results:** A total of 1,097 articles were initially identified, of which 28 studies involving 11,041 patients met the eligibility criteria for the meta-analysis. The pooled hazard ratios (HRs) for overall survival (OS) and disease-free survival (DFS) were 1.77 (95% *CI*: 1.53–2.05, *P* < 0.001) and 1.59 (95% *CI*: 1.47–1.71, *P* < 0.001), respectively, suggesting a significant correlation between the increased APRI levels and poor prognosis in the patients with HCC. In the subgroup analyses, statistical significance of the correlation disappeared in the Korean and Japanese population and in the patients undergoing transarterial chemoembolization (TACE). Of note, the current results may be overestimated due to publication bias, but the conclusion remained unchanged when the bias was adjusted.

**Conclusion:** High APRI levels are associated with poor OS and DFS in the patients with HCC. In most cases, pretreatment APRI can be used as an independent prognostic factor, but it is necessary to incorporate other predictive prognostic systems to ensure accuracy. Further studies are needed to determine the specific beneficiary population and the optimal cutoff value.

## Introduction

Hepatocellular carcinoma (HCC) is the third leading cause of cancer-related mortality worldwide with a poor 5-year survival rate (1). Despite the development of effective anti-viral therapeutics, incidence of HCC is continuing to rise, in part driven by the epidemic of non-alcoholic fatty liver disease (2). At present, in addition to liver transplantation, the surgical resection and chemotherapy are still the effective and routine options for HCC; accurate prognostic judgment is helpful for patient management and formulation of the medication strategies. Although the Tumor, Node, Metastasis (TNM) staging system is an effective independent prognostic factor for HCC, its predictive ability is lagging and limited. Therefore, it is necessary to combine new indices to improve the accuracy and timeliness of predicting survival in the patients with HCC.

Several inflammation-based markers and biomarkers of liver fibrosis and cirrhosis have been explored to predict prognosis (3). The previous studies have found that liver fibrosis and cirrhosis are important risk factors for postoperative recurrence of liver cancer (4–6). Liver biopsy is the gold standard for grading liver fibrosis or cirrhosis, but the invasive and expensive properties limited its clinical application (7). In 2003, aspartate aminotransferase (AST)-to-platelet (PLT) index (APRI) was first proposed by Wai et al. as a substitute index for the diagnosis of advanced fibrosis and cirrhosis in the patients with chronic hepatitis C; compared with liver biopsy, APRI is a simple, feasible, and non-invasive predictor and is derived from routine laboratory data calculated as the ratio of [(aspartate aminotransferase/upper limit of normal value)/platelet counts (× 10^9^/L)] × 100 (8–10). Meta-analysis has revealed that increased APRI levels can predict the risk of HCC development in the patients with chronic hepatitis (11). Furthermore, the preoperative APRI scores are associated with the postoperative complications of HCC and can predict the occurrence of hepatic failure after liver resection (12–15). As there is a strong correlation between APRI and liver histopathology, and liver fibrosis and cirrhosis are the key factors in the development of HCC; multiple studies attempt to explore the accuracy of APRI in predicting the prognosis of HCC.

The studies that explored the association between the APRI levels and the prognosis of HCC have shown inconsistent findings. In addition, these studies tend to come from a single center and are limited to specific populations, such as the patients with hepatitis B virus (HBV)-associated HCC or after hepatectomy. Therefore, to examine the prognostic potential of APRI comprehensively and systematically in the patients with HCC, we extensively reviewed all the cohort studies that explored the association of APRI levels with overall survival (OS) and disease-free survival (DFS) in the patients with HCC. Furthermore, we investigated the effects of multiple host and external factors on the predictive strength, which can help the clinicians fully utilize clinical data for risk stratification and develop personalized monitoring strategies.

## Methods and Materials

This meta-analysis was reported following the Preferred Reporting Items for Systematic Reviews and Meta-analyses (PRISMA 2020) statement (16). The protocol for this study is not registered.

### Search Strategy

Two reviewers independently conducted an extensive search of PubMed, Embase, and Web of Science databases to determine all relevant literature on November 15, 2020 and updated on June 20, 2021. The search query was formed by combining the subject words with free words, specifically, that include the following terms: “Carcinoma, Hepatocellular,” “Hepatocellular Carcinoma(s),” “Liver Cancer(s),” “Cell Carcinoma(s), Liver,” “Carcinoma(s), Liver Cell,” “Liver Cell Carcinoma(s),” “Adult Liver Cancer(s),” “Cancer(s), Adult Liver,” “Liver Cancer(s), Adult,” “Hepatoma(s),” “APRI,” “Aspartate aminotransferase-to-platelet ratio index,” and “AST/platelet ratio index.” There were no language restrictions or filters for the search. We also checked the reference lists of relevant reviews and included articles and performed a forward search. The search results discrepancies were resolved by consulting a library search professional.

The complete search strategies for each database are available in the Appendix.

### Eligibility Criteria

The included studies must meet all the following inclusion criteria: (1) the population was patients with HCC; (2) the subjects were divided into low and high APRI level groups; (3) the study investigated the hazard ratio (*HR*), risk ratio (*RR*), or odds ratio (*OR*) of OS or DFS between the different APRI levels or provided sufficient data to calculate theses risk effects; (4) the study type was a cohort study. The studies were excluded if it met any of the following criteria: (1) pathological type was not HCC; (2) not relevant to the prognosis of HCC; (3) the risk effects and corresponding 95% *CI* were not provided and available data did not enough to calculate them; (4) studies without original clinical data, such as the reviews, systematic reviews, meta-analysis, expert opinions, editorial, or comment. (5) The articles were not written in full text or the full text was not available, such as the conference abstract. If a patient cohort was included in multiple publications, we selected the study with the largest sample size among the eligible studies to avoid data overlap.

### Study Selection and Data Extract

Two authors independently reviewed the titles and abstracts of literature according to the eligibility criteria, initially excluding obvious irrelevant literature, and then re-evaluated the full text of the remaining articles to identify the relevant studies; the reasons for exclusion were recorded. Any disagreements were resolved through the discussion among all the authors. Two reviewers independently extracted the following data from the included literature: first author, publication year, sample size, gender, study region, median/mean age, follow-up time, treatment method, cutoff value of APRI, method of APRI selection, study design, proportion of patients with cirrhosis, vascular invasion, tumor size, number, and stage, hepatitis virus status, and the risk effects of OS and DFS along with corresponding 95% *CI* between high and low APRI groups. Considering that DFS and recurrence-free survival are often similar in clinical practice, we did not distinguish their *HR*s. Two reviewers cross-check the extracted data. If the study did not directly provide the risk effect and corresponding 95% *CI* but presented it as a figure, we extracted them using Engauge Digitizer software (version 4.1). In studies that provided both adjusted and unadjusted HRs, adjusted HR was preferred.

### Statistical Analysis

All data analysis was performed using Stata 14 (StataCorp LLC, TX, USA). We calculated the pooled *HR*s and corresponding 95% *CI*. Cochran's *Q*-test and Higgins' *I*^2^ statistics were used to assess the heterogeneity between the studies. When *I*^2^ < 50% and *P*_heterogeneity_ > 0.05, we considered that the heterogeneity was acceptable, and the fixed effects model was used to pool the results; otherwise, significant heterogeneity existed, and the random-effects model was selected (17). A univariate meta-regression analysis was performed to identify the potential sources of heterogeneity. The subgroup analyses were performed for the region, age (mean/median year <55 or ≥55), treatment, disease stage (CTP A, BCLC stage 0-A, Edmonson grade I–II, TNM stage I classified as early stage, and otherwise as advanced stage), hepatitis virus status, APRI level (cutoff value <1 or ≥1), method of APRI selection, proportion of cirrhosis (<50% or ≥50%), analysis mode, sample size (<300 or ≥300), and tumor size (median/mean size <3 cm or ≥3 cm). Hepatitis viral status and tumor stage were grouped according to the main patient characteristics of each study. The sensitivity analysis was performed by excluding one study at a time to evaluate the stability of the results. Potential publication bias was examined by visually inspecting the funnel plot and further confirmed by the Begg's and Egger's tests. No potential publication bias existed if both the *P*-values were >0.05 and the funnel diagram was symmetrical (17). When publication bias occurs, we use the trim-and-fill method to evaluate the influence of potentially unpublished studies on the pooled results (18). If *HR* < 1, APRI was considered a protective predictor of OS and DFS in the patients with HCC, while *HR* > 1 was considered a risk predictor. The *P*_overalleffect_ value <0.05 was considered statistically significant. The Newcastle-Ottawa Scale (NOS) scale (cohort study) was used to assess the quality of the included studies that mainly included selection, comparability, and outcome. When the NOS score ≥ 7, the study was classified as high quality, otherwise considered to be at high risk of bias (19).

## Results

**Figure 1:**
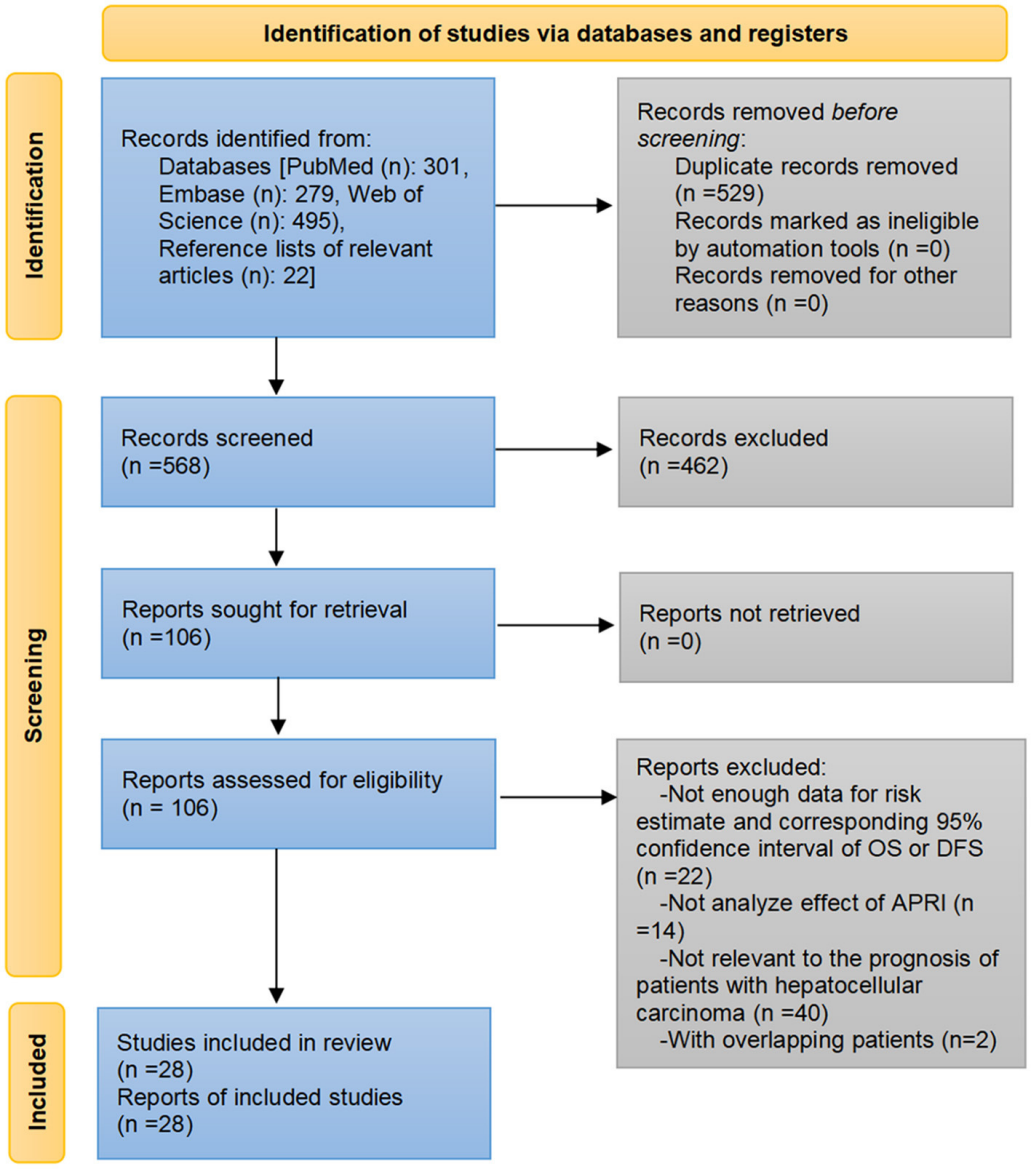
Flow diagram of the article selection process.

Through the pre-established search strategies, a total of 1,097 records were identified from Embase, PubMed, Web of Science, and reference lists of related papers. Eliminating duplicate literature and screening the titles and/or abstracts resulted in 991 records being excluded. We read the full text of the remaining 106 articles. Among them, 22 articles did not provide the risk effects along with corresponding 95% *CI* for OS or DFS and the data were insufficient to calculate them, 14 articles did not investigate the effect of APRI levels or analyze APRI separately, 40 articles were not relevant to the prognosis of HCC, two articles included the patients who overlapped with other larger cohorts. The list of articles excluded after a full-text reading is available in the Appendix. Finally, a total of 32 cohorts from 28 studies were included in the meta-analysis (20–47) (Figure 1).

### Description of the Included Studies

All the included 28 studies were retrospective cohort studies with a sample size ranging from 72 to 1,669, such as a total of 11,041 patients consisting of 8,776 men, 2,159 women, and 106 with unknown gender. Men account for the main proportion in all the studies. The median/mean age of patients in 11 studies was <55 years, 15 studies were >55 years, and the remaining two were unclear. The patients in 18 cohorts were treated with surgery, and the other cohorts included the patients with transarterial chemoembolization (TACE) (*n* = 4), radiotherapy (*n* = 1), radiofrequency ablation (RFA) (*n* = 3), or mixed therapy (*n* = 6), with a median/mean follow-up of 19.8–77.9 months. Some proportions or the entirety of patients in all the studies that reported hepatitis virus status, except one, were complicated with HBV (15.3–100%) and/or HCV (0.9–100%). Similarly, a certain percentage of patients in each evaluated study had cirrhosis (17.9–100%). Most of the studies were dominated by the patients with an early-stage and solitary number of tumors, and the proportion of patients with tumor vascular invasion ranged from 9.8 to 76.3%. The mean/median tumor size in 14 studies exceeded 3 cm. Thirteen studies were grouped with the APRI cutoff values <1, and 12 were ≥1. The APRI test timing in all the studies was before HCC treatment. The determination of the cutoff value was mainly based on an ROC analysis, as well as X-tile plots and other methods, such as experience or reference to the previous research. More detailed characteristics of the included studies are shown in Table 1. The NOS scores of the included studies ranged from 7 to 9, and the main risk of bias was short follow-up time (Table 2).

**Table 1:** The characteristics of the included studies.

**References**	**Region**	**No. (M/F)**	**Age (years)**	**Cutoff value**	**Cutoff value determination**	**Endpoint**	**Vascular invasion**	**Tumor size (cm)**	**Tumor number (multiple)**	**Proportion of cirrhosis**	**Hepatitis virus status**	**Tumor stage**	**Treatment**	**Follow-up (months)**
Zhang et al. (20)	China	405(356/49)	Median 52	0.45	ROC analysis	OS	NA	Median 4.8 (0.8–26)	14.8%	79.8%	HBV 100%	CTP: A (96.0%)–B (4.0%)	Surgery	Median 60.7 (36–117.8)
Lai et al. (21)	China	72(61/11)	Median 57	0.47	ROC analysis	OS, DFS	NA	Median 3.4 (1.5–5.0)	NA	NA	HBV 100%	CTP: A (87.5%)–B (12.5%)	Radiotherapy	Median 66.3 (7.6–125.8)
Zhao et al. (22)	China	429 (392/37)	Median 54	1.37	ROC analysis	OS	NA	NA	NA	NA	NA	CTP: A (59.0%)–B (32.9%)–C (8.1%)	Mixed	1–82.2
Zhao et al. (22)	China	169 (151/18)	Median 52	1.37	ROC analysis	OS,	NA	NA	NA	NA	NA	CTP: A (52.7%)–B (33.7%)–C (13.6%)	Mixed	1–82.2
Zhao et al. (22)	China	150 (131/19)	Median 48	1.37	ROC analysis	OS	NA	NA	NA	NA	NA	CTP: A (88.7%)–B (10.7%)–C (0.6%)	Surgery	1–82.2
Lee et al. (23)	Korea	184 (147/37)	Mean 52.3	1.5	NA	OS	36.4%	Median <3	7.1%	100%	HBV 100%	BCLC stage 0–A	Surgery	Mean 77.9
Li et al. (24)	China	628 (526/102)	Median 49.2	0.5	NA	OS, DFS	20.7%	Median 5.0	3.2%	75.3%	HBV 84.1%; HCV 2.1%	BCLC stage 0–A	Surgery	Mean 51.1 ± 31.8
Maegawa et al. (25)	USA	475 (361/8/ missing)	Mean 65.6	1.5	NA	OS	NA	NA	NA	NA	HCV 64.8%	CTP: A (72.8%)–B (27.2%)	Surgery	Mean 56.4 ± 45.6
Sonohara et al. (26)	Japan	305 (245/60)	Median 67	1.5	NA	OS, DFS	27.9%	Median 3.5 (0.1–21)	22.3%	41.2%	HBV 27.2%; HCV 45.6%	CTP: A (87.5%)–B (12.5%)	Surgery	Median 44 (0–188)
Yang et al. (27)	China	661 (574/87)	Mean: 47.45	0.25	ROC analysis	OS, DFS	NP	Median >5	29.7%	83.2%	HBV 85.5%	BCLC stage 0–A (35.7%)/B–C (64.3%)	Surgery	1–60
Matsumoto et al. (28)	Japan	162 (138/24)	Median 63	0.45	ROC analysis	OS, DFS	24.7%	Median 3.5	18.5%	43.2%	HBV: 27.8%; HCV:40.7%	CTP: A (92.6%)–B (7.4%)	Surgery	1–120
Sarkar et al. (29)	USA	94 (71/23)	Mean 62	0.5	NA	OS^*^	NA	Mean 2.3 ± 0.5	NA	91.5%	HBV: 30.9%; HCV: 55.3%	Single HCC ≤ 3.0 cm	Mixed	1–60
Allenson et al. (30)	USA	829 (645/184)	Mean 55.9	NA	NA	OS	NA	NA	NA	NA	HBV: 16.2%; HCV: 66.7%	Stage I (18.3%)–II (16.4%)–III (26.5%)–IV (33.2%)–unknown (5.4%)	Mixed	NA
Ji et al. (31)	China	321 (285/36)	Mean 51	1.68	NA	OS, DFS	NA	Median size > 5	30.0%	78.8%	HBV: 87.5%	Edmonson grade I–II (77.3%)/III–IV (22.7%)	Surgery	1–96
Zhu et al. (32)	China	351 (299/52)	Median age <65	0.5	X-tile plots	DFS	NA	Median size <5	13.7%	76.4%	HBV: 82.6%; HCV:1.4%	BCLC stage 0 (54.4%)–A (35.0%)–B (10.5%)	Surgery	Median 40.5
Tang et al. (33)	China	158 (136/22)	Median age ≤ 50	0.4	X-tile plots	OS^*^	NA	Median >5	>80.4%	NA	HBV: 93.7%	BCLC stage B	TACE	1–40
Tang et al. (33)	China	157 (135/22)	Median age ≤ 50	0.4	X-tile plots	OS^*^	NA	Median >5	>79.0%	NA	HBV: 92.4%	BCLC stage B	TACE	1–35
Chen et al. (34)	China	349(209/140)	Mean 66.9	1	NA	OS, DFS^*^^,^ ^#^	NA	Mean 1.8 ± 0.6^‡^	26.9%^‡^	NA	HBV: 37.0%; HCV: 59.6%	BCLC stage: 0 (54.2%)–A (45.8%)	RFA	Median 36.2
Jaruvongvanich et al. (35)	USA	900(660/240)	Mean 63.2	0.5, 1.5	NA	OS, DFS	NA	Median 2–5	31.6%	66.6%	HBV: 25.9%; HCV: 40.6%	BCLC stage: 0 (4.4%)–A (37.2%)–B (41.4%)–C (10.7%) -D (6.2%)	Mixed	Median 19.8
Shen et al. (36)	China	332 (292/40)	Mean 49.82	0.62	ROC analysis	OS, DFS	25.6%	Median >5	22.0%	76.2%	HBV: 85.5%	Edmonson grade I–II (77.4%)/III–IV (22.6%)	Surgery	1–80
Hung et al. (37)	China	76 (64/12)	Median 57	0.47	ROC analysis	OS, DFS^*^	76.3%	Median 2.5	NA	NA	HBV 100%	CTP: A (90.8%)–B (9.2%)	Surgery	Median 77.0 (4.7–226.6)
Toyoda et al. (38)	Japan	1,669 (1,181/488)	Mean 68.7	1.2	ROC analysis	OS, DFS^*^	16.90%	Median 2–5	38.60%	NA	HBV: 15.3%; HCV: 67.4%	BCLC stage 0 (15.6%)–A (43.8%)–B (12.2%)–C (18.4%)–D (9.2%)	Mixed	1–240
Choi et al. (39)	Korea	303 (246/57)	Median 55	NA	NA	OS^*^, DFS^*^	NA	3.7 (2.5–5.2	13.9%	49.8%	HBV	AJCC: I (64.0%)–II (32.3%)–IIIA (3.6%)	Surgery	Median 56.0
Okamura et al. (40)	Japan	140 (115/25)	Median 71	0.544	ROC analysis	OS^*^, DFS^*^	20.0%	Median 5.0 (1.0–17.5)	17.1%	17.9%	Non-HBV/HCV	CTP: A (97.9%)–B (2.1%)	Surgery	Median 38.9 (2.4–120)
Chung et al. (41)	Korea	98 (70/28)	Mean 60.5	1.38	ROC analysis	DFS	NA	Mean 1.9	NA	91.8%	HBV: 72.4%; HCV: 11.2%	CTP: A (81.6%)–B (18.4%)	RFA	Median 40 (4–95)
Kao et al. (42)	China	190 (121/69)	Mean 67.4	1	NA	OS, DFS	NA	Mean 2.4 ± 0.92	20.0%	NA	HBV: 47.6%; HCV: 45.7%	BCLC stage: 0 (15.3%)–A (75.8%)–B (8.9%)	RFA	Median 30.7 ± 17.5
Huang et al. (43)	China	451 (383/68)	Median age <60	0.9	X-tile plots	OS, DFS	30.4%	Median <5	2.9%	57.0%	HBV: 89.8%	BCLC stage: 0 (10.9%)–A (89.1%)	Surgery	3–86
Pang et al. (44)	China	172 (139/33)	Mean 53.5	1.23	ROC analysis	OS, DFS	31.4%	Median >5	18.6%	34.3%	HBV: 70.3%; HCV: 4.7%	CTP: A/B (93.0%)–C (7.0%)	Surgery	Median 46
Pang et al. (44)	China	191 (159/32)	Mean 54.1	1.79	ROC analysis	OS, DFS	49.7%	Median >5	28.8%	44.0%	HBV: 77.5%; HCV: 3.1%	CTP: A/B (86.4%)–C (13.6%)	TACE	Median 40
Liu et al. (45)	China	223 (189/34)	Median 54	0.23	ROC analysis	DFS^¶^	36.3%	Median >5	24.7%	89.2%	HBV: 78.0%; HCV: 0.9%	BCLC stage: 0/A (56.5%)-B/C (43.5%)	Surgery	Median 26.1 (1.9–72.6)
Peng et al. (46)	China	244 (213/31)	Mean 50	1	ROC analysis	OS, DFS	18.9%	Median 3–5	14.8%	85.7%	HBV: 96.3%	CTP A	Surgery	Median 36.3 (3–85.9)
Teng et al. (47)	China	153 (82/71)	Median 64.1	2	NA	OS, DFS	9.8%	Median 2.7 (1.9–3.8)	NA	NA	HCV: 100%	BCLC stage 0 (4.6%)–A (30.7%)–B (31.4%)–C (33.3%)	TACE	1–60

*M/F, male/female; CTP, Child-Turcotte-Pugh; AJCC, American Joint Committee on Cancer TNM staging system; TACE, transarterial chemoembolization; RFA, radiofrequency ablation; BCLC, Barcelona Clinic Liver Cancer; DFS, disease-free survival; HBV, hepatitis B virus; HCV, hepatitis C virus; NA, not available; ROC, receiver operating characteristic curve*.

* *Univariate analysis*.

# *Second recurrence*.

‡ *Recurrent*.

¶ *Including early recurrence and late recurrence analysis*.

**Table 2:** The quality assessment of the included studies.

**References**	**Representativeness of exposed cohort**	**Selection of non-exposed cohort**	**Ascertainment of exposure**	**Outcome not present before study**	**Comparability**	**Assessment of outcome**	**Follow-up long enough^¶^**	**Adequacy of follow up**	**Quality score**
Zhang et al. (20)	⋆	⋆	⋆	⋆	⋆⋆	⋆	⋆	⋆	9
Lai et al. (21)	⋆	⋆	⋆	⋆	⋆⋆	⋆	⋆	⋆	9
Zhao et al. (22)	⋆	⋆	⋆	⋆	⋆⋆	⋆		⋆	8
Lee et al. (23)	⋆	⋆	⋆	⋆	⋆⋆	⋆	⋆	⋆	9
Li et al. (24)	⋆	⋆	⋆	⋆	⋆⋆	⋆		⋆	8
Maegawa et al. (25)	⋆	⋆	⋆	⋆	⋆⋆	⋆		⋆	8
Sonohara et al. (26)	⋆	⋆	⋆	⋆	⋆⋆	⋆		⋆	8
Yang et al. (27)	⋆	⋆	⋆	⋆	⋆⋆	⋆		⋆	8
Matsumoto et al. (28)	⋆	⋆	⋆	⋆	⋆⋆	⋆	⋆	⋆	9
Sarkar et al. (29)	⋆	⋆	⋆	⋆	⋆	⋆		⋆	7
Allenson et al. (30)	⋆	⋆	⋆	⋆	⋆⋆	⋆			7
Ji et al. (31)	⋆	⋆	⋆	⋆	⋆⋆	⋆		⋆	8
Zhu et al. (32)	⋆	⋆	⋆	⋆	⋆⋆	⋆		⋆	8
Tang et al. (33)	⋆	⋆	⋆	⋆	⋆	⋆		⋆	7
Chen et al. (34)	⋆	⋆	⋆	⋆	⋆	⋆		⋆	7
Jaruvongvanich et al. (35)	⋆	⋆	⋆	⋆	⋆⋆	⋆		⋆	8
Shen et al. (36)	⋆	⋆	⋆	⋆	⋆⋆	⋆		⋆	8
Hung et al. (37)	⋆	⋆	⋆	⋆	⋆	⋆	⋆	⋆	8
Toyoda et al. (38)	⋆	⋆	⋆	⋆	⋆	⋆	⋆	⋆	8
Choi et al. (39)	⋆	⋆	⋆	⋆	⋆	⋆		⋆	7
Okamura et al. (40)	⋆	⋆	⋆	⋆	⋆	⋆	⋆	⋆	8
Chung et al. (41)	⋆	⋆	⋆	⋆	⋆⋆	⋆		⋆	8
Kao et al. (42)	⋆	⋆	⋆	⋆	⋆⋆	⋆		⋆	8
Huang et al. (43)	⋆	⋆	⋆	⋆	⋆⋆	⋆		⋆	8
Pang et al. (44)	⋆	⋆	⋆	⋆	⋆⋆	⋆		⋆	8
Liu et al. (45)	⋆	⋆	⋆	⋆	⋆⋆	⋆		⋆	8
Peng et al. (46)	⋆	⋆	⋆	⋆	⋆⋆	⋆		⋆	8
Teng et al. (47)	⋆	⋆	⋆	⋆	⋆⋆	⋆		⋆	8

¶ *The median/mean follow-up time ≥60 months or the longest follow-up time ≥120 months is acceptable*.

### Association Between the APRI Levels and OS

**Figure 2:**
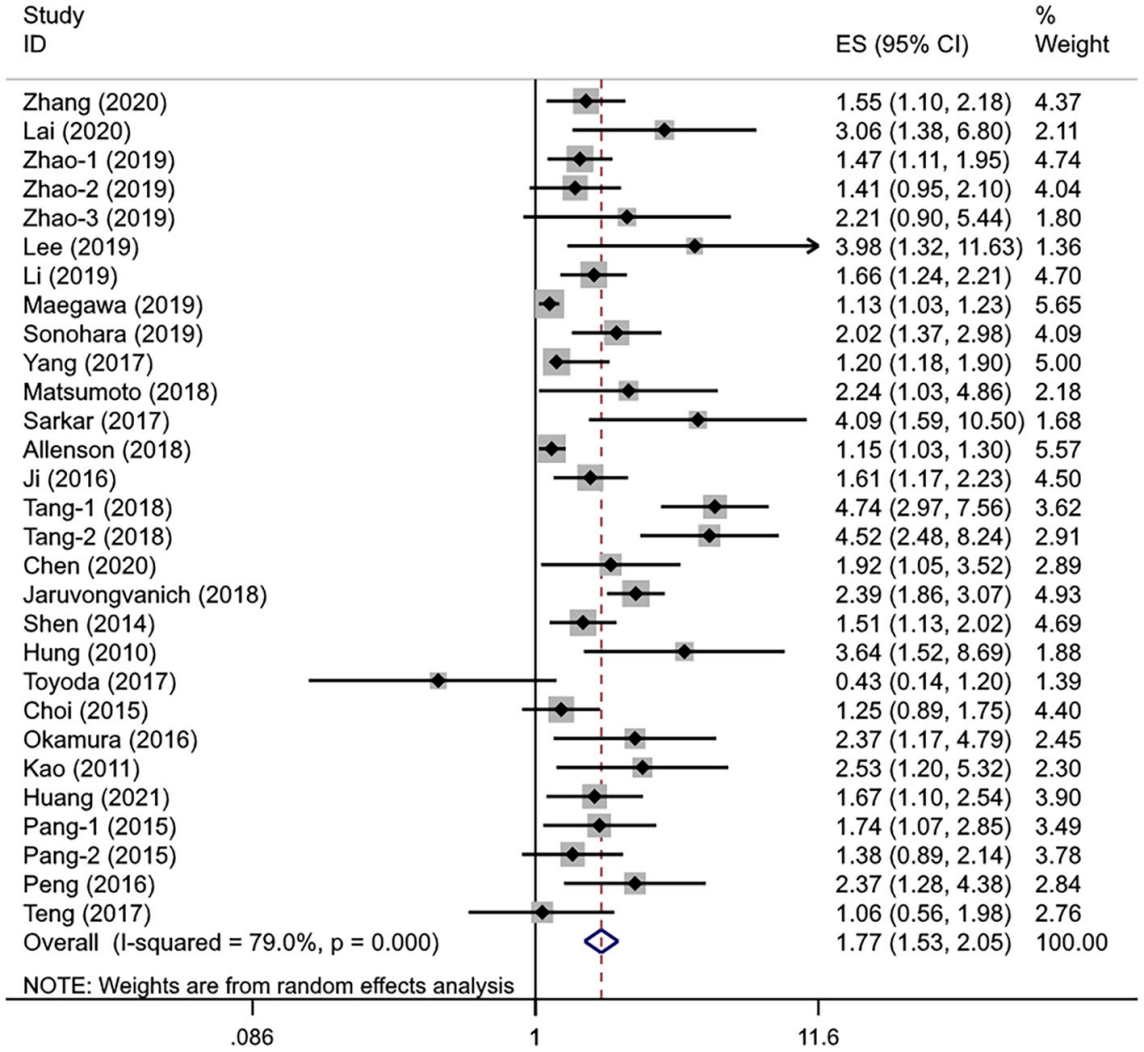
The forest plot of pooled hazard ratio (*HR*) for overall survival (OS).

A total of 25 studies involving 10,369 patients with HCC evaluated the association between different APRI levels and OS. The heterogeneity test showed significant heterogeneity (*I*^2^ = 79.0%, *P*_heterogeneity_ < 0.001), so the random-effects model was used for effect size analysis. The pooled *HR* was 1.77 (95% *CI*: 1.53–2.05, *P*_overall effect_ < 0.001), indicating that the APRI index had a significant predictive ability for the OS of patients with HCC (Figure 2). The patients with high APRI levels had an increased risk of death by 77% compared with the patients with low APRI levels.

In the subgroup analysis and regression analysis, we found that the sample size and analysis mode were the possible source of heterogeneity (*P*_interaction_ = 0.003 and 0.017, respectively); the studies with fewer than 300 subjects and the univariate analysis reported a higher *HR*. Furthermore, APRI cutoff value (*P*_interaction_ = 0.083) and cutoff determination method (*P*_interaction_ = 0.094) may also be the sources of heterogeneity, while age, treatment, study region, disease stage, hepatitis virus status, cirrhosis, and tumor size were not related to observed substantial heterogeneity. A multivariate analysis showed that APRI could be an independent predictive factor for the OS (*HR* =1.60, 95% *CI*: 1.40–1.83, *P*_overall effect_ < 0.001). Of note, the pooled results from the studies in Japan (*HR* = 1.67, 95% *CI*: 0.95–2.93, *P*_overall effect_ = 0.076) and Korea (*HR* = 1.98, 95% *CI*: 0.65–6.00, *P*_overall effect_ = 0.229) showed no significant correlation between APRI and OS. In other subgroups, higher APRI levels were consistently associated with poorer OS (Table 3). In addition, we performed a univariate meta-regression analysis based on the sex ratio of the patients in each study, and no statistical significance was observed (*P* = 0.202).

**Table 3:** The results of the subgroup analysis for overall survival (OS).

**Subgroups**	**No. of studies**	**Pooled HR (95%CI)**	***P*_**overall effect**_**	**Heterogeneity(*I*^**2**^, *P*_**Q**_)**	***P*_**interaction**_**
**Median/mean age**					
<55 years	10	1.83 (1.50–2.23)	<0.001	70.4%, <0.001	0.695
≥55 years	14	1.70 (1.37–2.12)	<0.001	81.4%, <0.001	
**Treatment**					
Surgery	16	1.63 (1.39–1.90)	<0.001	65.4%, <0.001	0.362
RFA	2	2.14 (1.34–3.43)	0.001	0.0%, 0.573	
TACE	3	2.38 (1.11–5.07)	0.025	88.0%, <0.001	
Radiotherapy	1	3.06 (1.38–6.80)	0.006	-	
Mixed	5	1.53 (1.06–2.20)	0.023	86.6%, <0.001	
**Region**					
China	15	1.83 (1.55–2.17)	<0.001	64.2%, <0.001	0.571
Korea	2	1.98 (0.65–6.00)	0.229	74.8%, 0.046	
USA	4	1.55 (1.13–2.12)	0.006	92.1%, <0.001	
Japan	4	1.67 (0.95–2.93)	0.076	62.4%, 0.046	
**Sample size**					
<300	12	2.39 (1.83–3.21)	<0.001	61.2%, 0.001	0.003
≥300	14	1.47 (1.27–1.69)	<0.001	76.8%, <0.001	
**Disease stage**					
Early	19	1.72 (1.46–2.01)	<0.001	66.9%, <0.001	0.646
Advanced	5	2.00 (1.28–3.11)	0.002	93.0%, <0.001	
**Hepatitis virus status**					
HBV	14	1.94 (1.59–2.37)	<0.001	70.9%, <0.001	0.459
HCV	9	1.55 (1.20–1.98)	0.001	84.8%, <0.001	
Non-HBV/HCV	1	2.37 (1.17–4.79)	0.016	-	
**APRI level**					
<1	11	2.22 (1.69–2.91)	<0.001	76.7%, <0.001	0.083
≥1	11	1.56 (1.29–1.89)	<0.001	64.1%, 0.001	
**APRI selection**					
ROC analysis	11	1.51 (1.35–1.69)	<0.001	40.2%, 0.060	0.094
X-tile plots	2	3.24 (1.58–6.67)	0.001	84.6%, 0.002	
Other	12	1.63 (1.34–1.99)	<0.001	81.8%, <0.001	
**Proportion of cirrhosis**					
<50%	5	1.62 (1.34–1.95)	<0.001	15.0%, 0.318	0.798
≥50%	10	1.76 (1.45–2.12)	<0.001	61.0%, 0.006	
**Analysis modes**					
Univariate analysis	4	2.96 (1.54–5.71)	0.001	71.8%, <0.001	0.017
Multivariate analysis	21	1.60 (1.40–1.83)	<0.001	85.7%, <0.001	
**Median/mean tumor size**					
<3 cm	6	2.18 (1.60–2.98)	<0.001	47.6%, 0.089	0.401
≥3 cm	13	1.89 (1.55–2.30)	<0.001	70.4%, <0.001	

*RFA, radiofrequency ablation; TACE, transarterial chemoembolization; HBV, hepatitis B virus; HCV, hepatitis C virus; ROC, receiver operating characteristic curve; APRI, aspartate aminotransferase-to-platelet ratio index*.

### Association Between the APRI Levels and DFS

**Figure 3:**
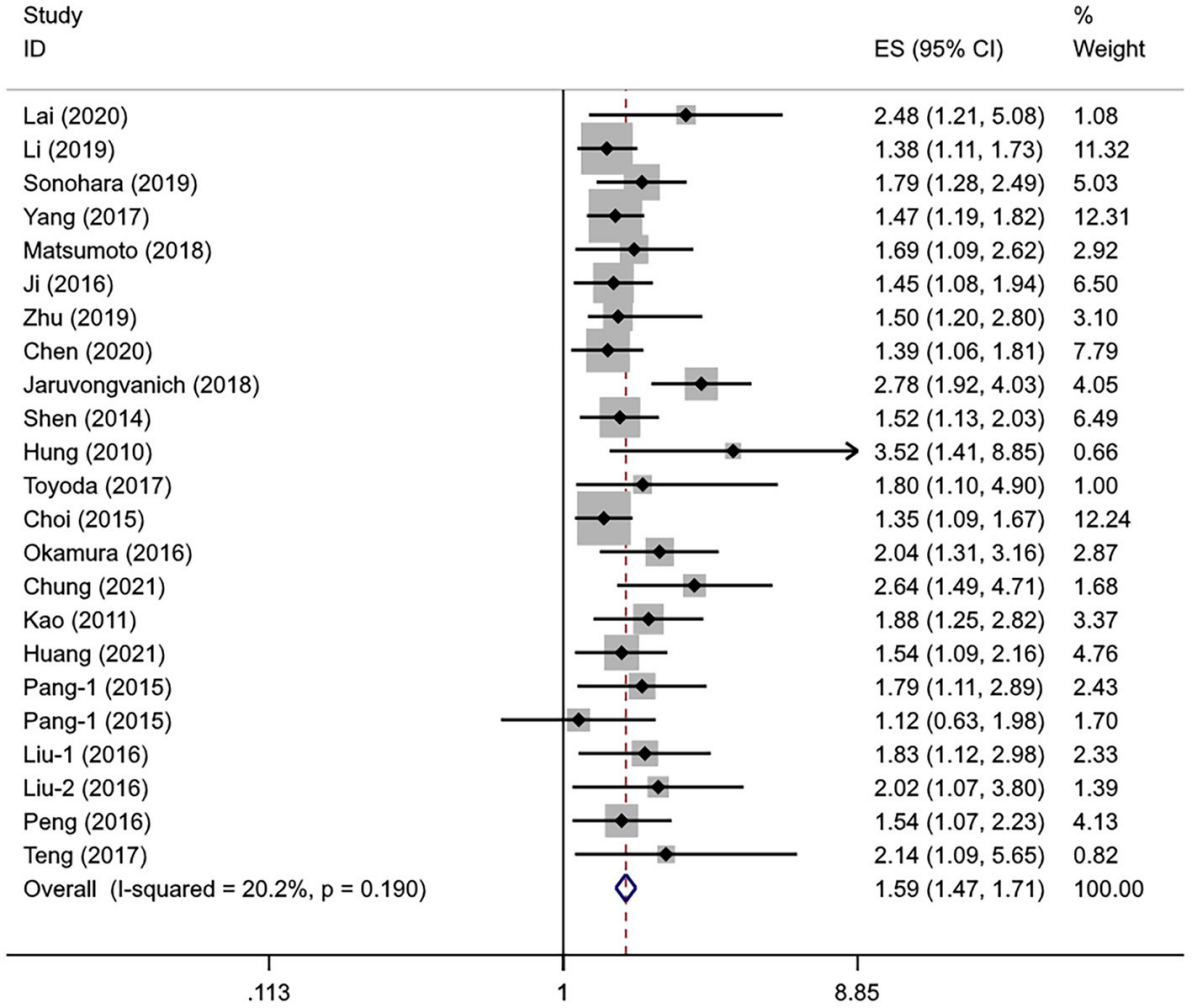
The forest plot of pooled *HR* for DFS.

A total of 21 studies with 7,991 patients with HCC evaluated the association between the APRI levels and DFS. The heterogeneity level was subtle (*I*^2^ = 20.2%, *P*_heterogeneity_ = 0.190), so the fixed-effects model was used. Similar to OS, the pooled *HR* showed a significant association between the higher APRI levels and poorer DFS (*HR* = 1.59, 95% *CI*: 1.47–1.71, *P*_overall effect_ < 0.001) (Figure 3).

The sample size remained the potential source of heterogeneity (*P*_interaction_ = 0.037); the studies with the small sample sizes tended to report a higher *HR* than the large sample sizes. Age (*P*_interaction_ = 0.084) and hepatitis virus status (*P*_interaction_ = 0.081) may also be the sources of heterogeneity. A subgroup analysis showed no statistical correlation between the APRI levels and DFS in the Korean population (*HR* = 1.79, 95% *CI*: 0.93–3.42, *P*_overall effect_ = 0.079) and those who received TACE (*HR* = 1.38, 95% *CI*: 0.86–2.21, *P*_overall effect_ = 0.176). The statistical significance could be consistently observed in other subgroups. The pooled results of the multivariate analysis indicated that APRI could serve as an independent predictor of DFS in the patients with HCC (*HR* = 1.62, 95% *CI*: 1.49–1.77, *P*_overall effect_ < 0.001) (Table 4). The regression analysis showed that the change of *HR* with sex ratio was not statistically significant (*P* = 0.168).

**Table 4:** The results of the subgroup analysis for disease-free survival (DFS).

**Subgroups**	**No. of studies**	**Pooled HR (95%CI)**	***P*_**overall effect**_**	**Heterogeneity (*I^**2**^*, *P*_**Q**_)**	***P*_**interaction**_**
**Median/Mean age**					
<55	7	1.49 (1.34–1.66)	<0.001	0.0%, 0.888	0.084
≥55	12	1.87 (1.58–2.22)	<0.001	46.1%, 0.040	
**Treatment**					
Surgery	14	1.53 (1.41–1.67)	<0.001	0.0%, 0.766	0.268
RFA	3	1.78 (1.25–2.51)	0.001	55.9%, 0.104	
TACE	2	1.38 (0.86–2.21)	0.176	37.6%, 0.205	
Radiotherapy	1	2.48 (1.21–5.08)	0.013	-	
Mixed	2	2.55 (1.83–3.56)	<0.001	4.2%, 0.307	
**Region**					
China	14	1.53 (1.40–1.67)	<0.001	0.0%, 0.757	0.357
Korea	2	1.79 (0.93–3.42)	0.079	78.2%, 0.032	
USA	1	2.78 (1.92–4.03)	<0.001	-	
Japan	4	1.82 (1.47-2.26)	<0.001	0.0%, 0.945	
**Sample size**					
<300	10	1.85 (1.59–2.14)	<0.001	0.0%, 0.670	0.037
≥300	11	1.51 (1.38–1.65)	<0.001	27.7%, 0.180	
**Disease stage**					
Early	17	1.56 (1.44–1.70)	<0.001	0.0%, 0.524	0.321
Advanced	3	2.01 (1.23–3.27)	0.005	77.3%, 0.012	
**Hepatitis virus status**					
HBV	14	1.52 (1.40–1.66)	<0.001	0.0%, 0.483	0.081
HCV	6	1.77 (1.51–2.08)	<0.001	45.0%, 0.106	
Non-HBV/HCV	1	2.04 (1.31–3.16)	0.002	-	
**APRI level**					
<1	10	1.57 (1.41–1.75)	<0.001	0.0%, 0.556	0.802
≥1	9	1.60 (1.41–1.82)	<0.001	0.0%, 0.558	
**APRI selection**					
ROC analysis	11	1.66 (1.48–1.87)	<0.001	0.0%, 0.498	0.394
X-tile plots	2	1.52 (1.17–1.99)	0.002	0.0%, 0.925	
Other	8	1.62 (1.37–1.90)	<0.001	54.0%, 0.033	
**Proportion of cirrhosis**					
<50%	5	1.54 (1.34–1.78)	<0.001	10.0%, 0.352	0.800
≥50%	10	1.59 (1.44–1.75)	<0.001	34.5%, 0.123	
**Analysis mode**					
Univariate analysis	5	1.48 (1.28–1.73)	<0.001	39.8%, 0.156	0.483
Multivariate analysis	16	1.62 (1.49–1.77)	<0.001	14.6%, 0.280	
**Median/mean tumor size**					
<3 cm	5	1.72 (1.41–2.10)	<0.001	45.6%, 0.118	0.273
≥3 cm	12	1.52 (1.39–1.66)	<0.001	0.0%, 0.723	

*RFA, radiofrequency ablation; TACE, transarterial chemoembolization; HBV, hepatitis B virus; HCV, hepatitis C virus; ROC, receiver operating characteristic curve; APRI, aspartate aminotransferase-to-platelet ratio index*.

### Sensitivity Analysis

**Figure 4:**
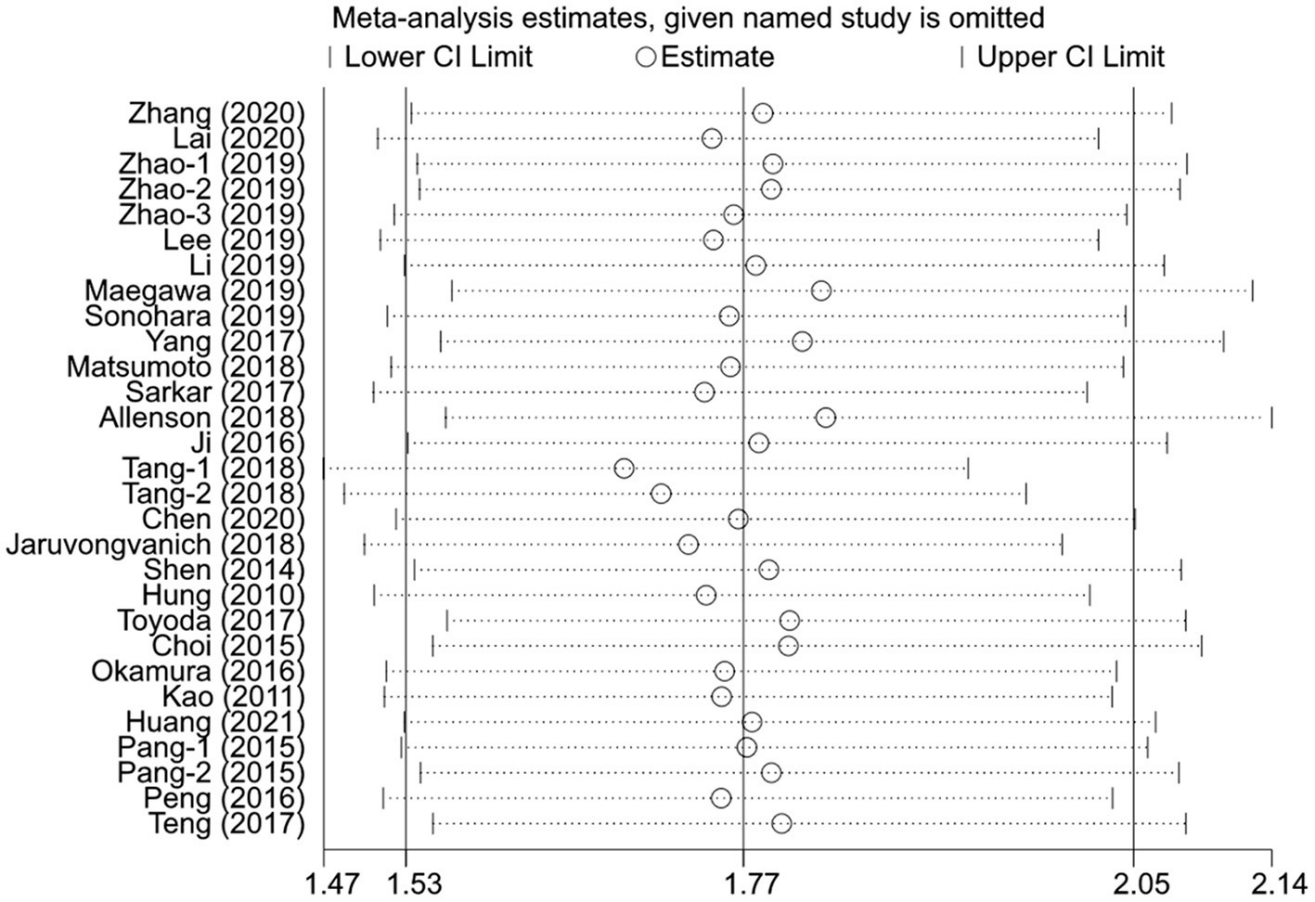
The effects of the individual studies on the pooled *HR* of OS.

**Figure 5:**
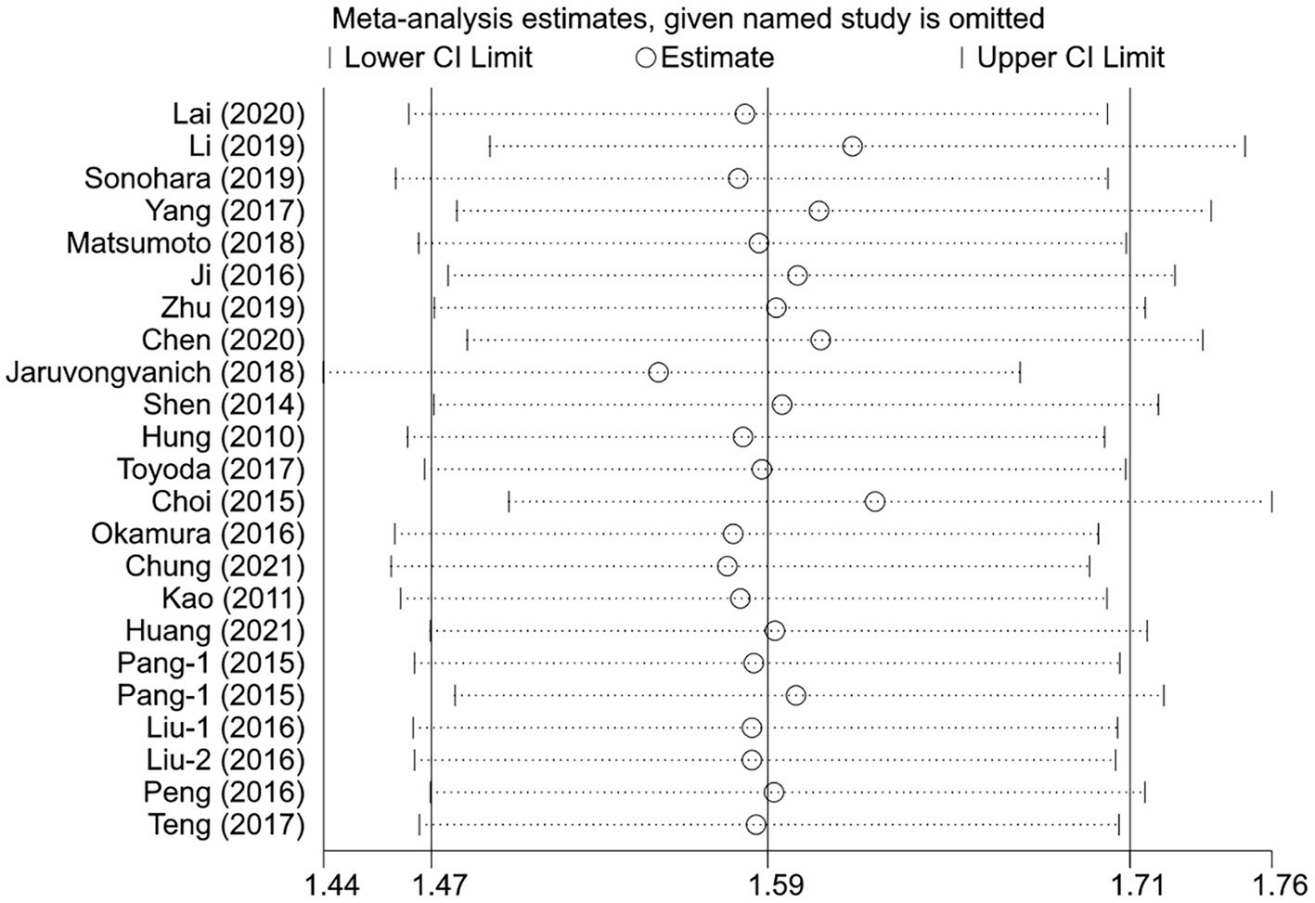
The effects of the individual studies on the pooled *HR* of DFS.

The sensitivity analysis was performed for the pooled results by excluding one study at a time. The results showed that the pooled effect sizes were stable, and each individual study had little effect on the pooled *HR*s for OS and DFS (Figures 4, 5).

### Publication Bias

**Figure 6:**
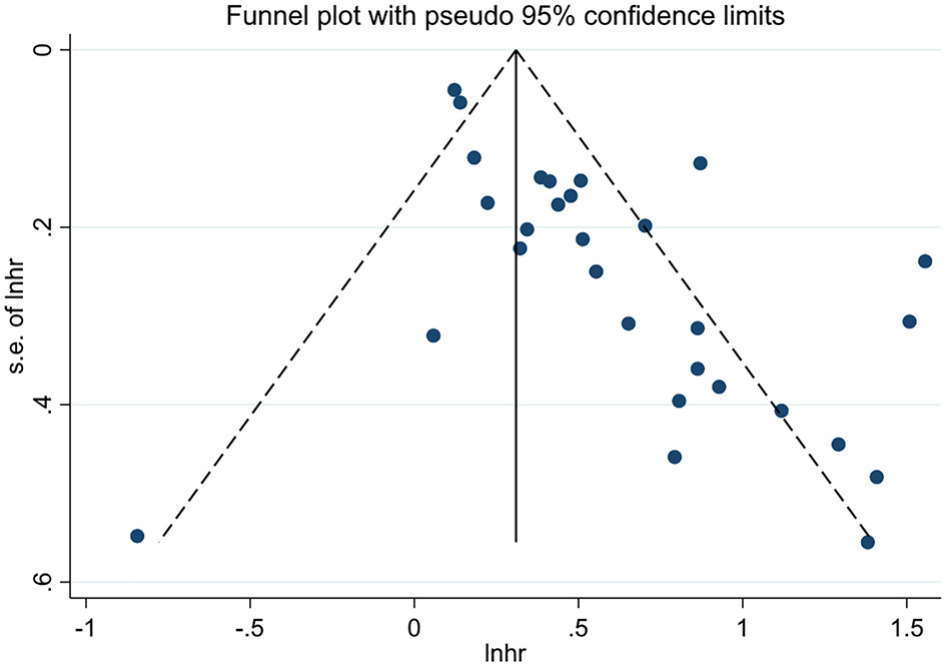
The funnel plot for OS.

**Figure 7:**
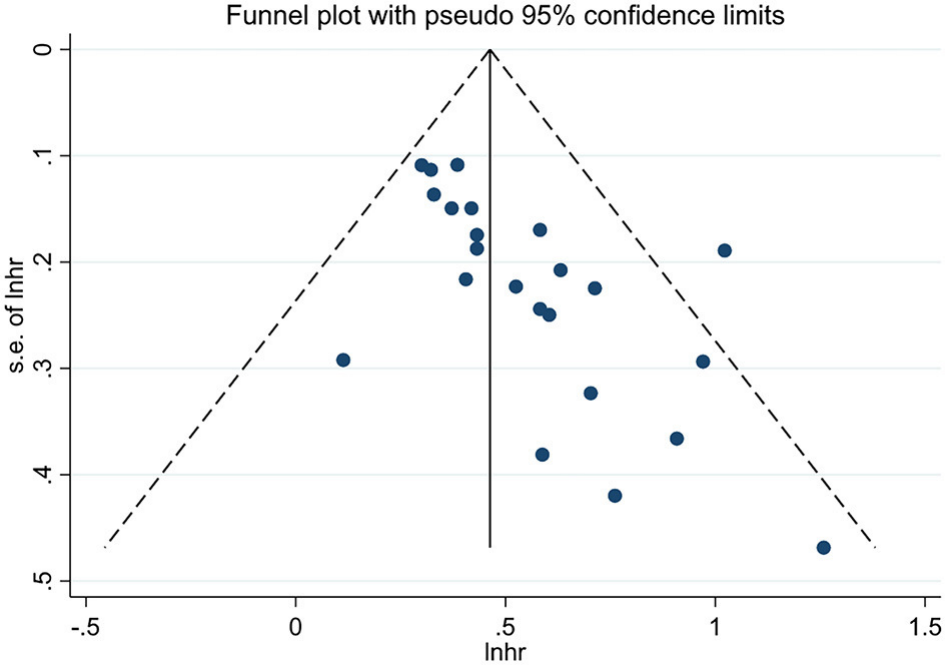
The funnel plot for DFS.

We performed the funnel plots to evaluate the publication bias of included 28 studies, and the results showed obvious asymmetry (Figures 6, 7). The Begg's and Egger's tests were performed to validate the results, and both the *P*-values for OS and DFS were <0.05, demonstrating the risk of publication bias in the current published studies. In OS analysis, after adjusting with the trim-and-fill method, 12 unpublished studies were added. The re-pooled results showed that the *HR*s of the fixed-effects model and the random-effects model were 1.25 (95% *CI*: 1.19–1.31, *P* < 0.001) and 1.32 (95% *CI*: 1.13–1.55, *P* < 0.001), respectively. In DFS analysis, eight unpublished studies were added according to the trim-and-fill method. The re-pooled results showed that the *HR*s of the fixed-effects model and the random-effects model were 1.48 (95% *CI*: 1.38–1.59, *P* < 0.001) and 1.50 (95% *CI*: 1.35–1.66, *P* < 0.001), respectively.

## Discussion

In this study, OS and DFS were used as the endpoints to evaluate whether the APRI levels could predict the prognosis of HCC. The overall pooled results suggested that the patients with HCC with high APRI levels had a significantly increased risk of death and recurrence than the patients with low APRI levels. Although the pooled results may be influenced by publication bias, the significant correlation between the APRI levels and prognosis of HCC remained after adding potentially unpublished literature. Furthermore, we analyzed the impacts of age, treatment, region, sample size, disease stage, hepatitis virus status, APRI cutoff value, cutoff value selection, cirrhosis, analysis mode, and tumor size on the results. Among them, the pooled results from Korea, Japan, and the patients treated with TACE showed no statistical association between the pretreatment APRI levels and prognosis (OS and/or DFS), while a stable correlation could be observed in other subgroups. The results from the multivariate analysis suggested that APRI levels seem to serve as an independent predictive factor for the patients with HCC.

In addition to predicting the prognosis of patients with HCC throughout the follow-up period, Liu et al. found that the APRI levels could be an independent risk factor for early recurrence <1 year in the background of cirrhosis/fibrosis, and the study conducted by Allenson et al. showed that a high APRI level was associated with OS <30 days (30, 45). Furthermore, Chen et al. found that a high APRI level predicted a higher risk of second recurrence (34). These findings can broaden the prediction range of APRI.

Lee et al. and Chung et al. also found that the patients who developed HCC death or relapses had persistently higher APRI values than the non-relapsing patients during follow-up (23, 41). Moreover, the study conducted by Peng et al. indicated that the difference value between postoperative APRI and preoperative APRI could serve as an independent prognostic factor for OS and DFS; change of APRI ≥ 0.02 was associated with the poorer outcomes (46). A similar result was found in the study conducted by Lee et al. (23). In addition, Chung et al. observed that the APRI value decreased significantly during follow-up in the patients who remained relapse-free, suggesting that it may be feasible to use APRI as a dynamic monitoring indicator during follow-up (41). However, there was a lack of data on dynamic AST and/or PLT, and no detailed description of the specific fluctuations of APRI over time.

APRI is a non-invasive serum predictor that can be calculated at the bedside using the routine laboratory test parameters. The exact mechanism between APRI and poor prognosis in the patients with HCC after treatment remains unclear. It may be explained by the following reasons. First, the PLT count can promote hepatocyte regeneration and reflect the degree of liver function; thrombopenia is considered a risk factor for cirrhosis, and the patients with cirrhosis generally have decreased the PLT levels due to reduced thrombopoietin and PLT capture by the liver (48–53). PLT is also an important regulator of tumor vascular homeostasis and can facilitate and regulate tumor angiogenesis (54). Second, AST not only represents the degree of cirrhosis, but is also a biological indicator of liver inflammation; as AST exists in the mitochondria of liver cells, it can be released into the serum when mitochondria are damaged, and therefore AST is used as a reliable and sensitive biomarker to indicate the damage of liver function (55, 56). Liver cirrhosis and hepatocyte damage were closely associated with hepatocarcinogenesis (57–59). As a result, APRI that combined AST and PLT count is expected to reflect the prognosis of HCC from liver reserve and inflammation. Several studies have suggested that the low preoperative PLT or the high AST levels are associated with poor survival after therapy (60–63).

Of note, although current data show that the APRI level can generally reflect the prognosis of patients with HCC, it cannot determine which populations can achieve the best sensitivity and specificity, so it is necessary to combine with other prognostic prediction systems in clinical practice. On the one hand, it can be combined with currently used clinical assessment methods, such as CT, PET-CT, MRI, DSA, ultrasonography, TNM staging system, AFP, or Model for End-Stage Liver Disease score. On the other hand, it is possible to combine other serum markers with prognostic value, such as AST/lymphocyte ratio, neutrophil/lymphocyte ratio, and platelet/lymphocyte ratio, to establish a novel prognostic scoring system. For instance, Zhao et al. established a prognostic nomogram involving APRI, AST/lymphocyte ratio, and systemic immune-inflammation index, and Ji et al. found that APRI combined with neutrophil/lymphocyte ratio improved the predictive accuracy (22, 23).

The main limitation of this study was the small number of studies in some subgroups (such as non-surgical treatment group, non-HBV/HCV HCC group, United States, European, and African region group), so it is difficult to draw solid conclusions that are universally applicable to all the regions or all populations. The absence of statistical significance in some subgroups could also be explained by a lack of statistical power due to the small number of included studies. Furthermore, the published studies did not provide sufficient data for a more detailed subgroup analysis to accurately characterize the population that can benefit most from the APRI prediction strategy or is unsuitable for applying APRI scoring. Next, all the included studies were retrospective studies that did not consider scientific research needs in the collection of clinical data and did not guarantee the completeness and homogeneity of data. Third, some factors that may affect the results, such as tumor differentiation, vascular invasion, and tumor number, were not included in the analysis due to a lack of detailed data. In addition, the APRI level was also affected by physical activity, anti-hepatitis virus drugs, and concomitant diseases. Therefore, it will be necessary to proceed with caution when formulating a prognostic risk assessment strategy based on these data. Fourth, although the studies with different APRI cutoff values have shown that high APRI can predict worse OS and DFS, it must be unified before APRI score is widely used in clinical settings. Finally, the current results may be impacted by publication bias. Although the conclusion remains unchanged after adding potentially unpublished studies, the presence of publication bias should be emphasized when determining the APRI optimal cutoff value and evaluating prognostic value in the specific patients.

## Conclusion

Although APRI is not yet suitable as a single independent prognostic factor for the patients with HCC, we suggest that the clinicians can use the APRI scores combined with other prognostic assessment systems for post-operative risk stratification, which may help to improve the predictive accuracy, leading to more effective clinical decision-making and prolonged survival of patients. Furthermore, more prospective studies with large samples and long-term follow-up from different regions and populations are needed to evaluate the prognostic value and optimal cutoff value of APRI in the patients with different characteristics.

## Data Availability Statement

The original contributions presented in the study are included in the article/Supplementary Material, further inquiries can be directed to the corresponding author/s.

## Author Contributions

QS contributed to the conception and design of the study. MLiv and ZS searched the database. XZ and MLiu extracted data. ZS and YZ performed the statistical analysis. XZ, MLiv, and ZS wrote the manuscript. ZS contributed to critical revision of the manuscript. All the authors approved the submitted version.

## Funding

The research was supported by the Talent Introduction and Research Start-up Fund of Southwest Medical University (00040056), the Joint Project of the Southwest Medical University-Affiliated Hospital of Traditional Chinese Medicine of Southwest Medical University (2018 No. 6-51), Sichuan Provincial Administration of Traditional Chinese Medicine Project (2021 No. 13-130), Science & Technology Department of Sichuan Provincial Project (2021YFH0150), and People's Government of Luzhou City-Southwest Medical University High-level Talent Introduction Special Funding Project (2017RC-002).

## Conflict of Interest

The authors declare that the research was conducted in the absence of any commercial or financial relationships that could be construed as a potential conflict of interest.

## Publisher's Note

All claims expressed in this article are solely those of the authors and do not necessarily represent those of their affiliated organizations, or those of the publisher, the editors and the reviewers. Any product that may be evaluated in this article, or claim that may be made by its manufacturer, is not guaranteed or endorsed by the publisher.
