# Supplementary material for: Assessment of Prognostic Value of Aspartate Aminotransferase-to-Platelet Ratio Index in Patients With Hepatocellular Carcinoma: Meta-Analysis of 28 Cohort Studies
Source: Front Med (Lausanne). 2021 Nov 26;8:756210. doi: 10.3389/fmed.2021.756210 (PMC8661594; doi:10.3389/fmed.2021.756210)
Supplement: Supplementary file 1 [file Data_Sheet_1.DOCX]

**Search strategies of each database:**

**PubMed:**

(aspartate aminotransferase-to-platelet ratio index OR aspartate aminotransferase/platelet count ratio index OR aspartate aminotransferase: platelet ratio index OR AST-to-platelet ratio index OR AST/ PLT ratio index OR APRI ) AND ((Carcinomas, Hepatocellular OR Hepatocellular Carcinomas OR Liver Cell Carcinoma, Adult OR Liver Cancer, Adult OR adult liver cancers or adults liver cancers OR Cancer, Adult Liver OR Cancers, Adult Liver OR Liver Cancers, Adult OR Liver Cell Carcinoma OR Carcinoma, Liver Cell OR Carcinomas, Liver Cell OR Cell Carcinoma, Liver OR Cell Carcinomas, Liver OR Liver Cell Carcinomas OR Hepatocellular Carcinoma OR Hepatoma OR Hepatomas) OR ("Carcinoma, Hepatocellular"[Mesh]))

**Web of science:**

TS=((Carcinomas, Hepatocellular OR Hepatocellular Carcinomas OR Liver Cell Carcinoma, Adult OR Liver Cancer, Adult OR adult liver cancers or adults liver cancers OR Cancer, Adult Liver OR Cancers, Adult Liver OR Liver Cancers, Adult OR Liver Cell Carcinoma OR Carcinoma, Liver Cell OR Carcinomas, Liver Cell OR Cell Carcinoma, Liver OR Cell Carcinomas, Liver OR Liver Cell Carcinomas OR Hepatocellular Carcinoma OR Hepatoma OR Hepatomas OR Carcinoma, Hepatocellular) and (aspartate aminotransferase-to-platelet ratio index OR aspartate aminotransferase/platelet count ratio index OR aspartate aminotransferase: platelet ratio index OR AST-to-platelet ratio index OR AST/ PLT ratio index OR APRI ))

Databases= All databases

**Embase:**

Sources: Embase, MEDLINE

Query: ('liver cell carcinoma'/exp OR 'carcinoma, hepatic cell' OR 'carcinoma, hepatocellular' OR 'carcinoma, liver' OR 'carcinoma, liver cell' OR 'hepatic carcinoma' OR 'hepatic cell carcinoma' OR 'hepatocarcinoma' OR 'hepatocellular carcinoma' OR 'hepatoma' OR 'liver carcinoma' OR 'liver carcinoma rupture' OR 'malignant hepatoma' OR 'primary liver carcinoma') AND ('aspartate aminotransferase to platelet ratio index'/exp OR 'aspartate amino transferase to platelet ratio index' OR 'aspartate aminotransferase/platelet ratio index' OR 'aspartate transaminase (ast) to platelet (plt) ratio index' OR 'aspartate transaminase (ast) to platelets ratio index' OR 'aspartate transaminase level to platelet count ratio index' OR 'aspartate transaminase to platelet ratio index' OR 'aspartate transaminase to platelets ratio index' OR 'aspartate transaminase to the platelet ratio index' OR 'aspartate transaminase/platelet ratio index' OR 'aspartic aminotransferase to platelet ratio index' OR 'ast to platelet ratio index':ab,ti OR 'ast to platelets ratio index' OR 'ast/platelet ratio index' OR 'ast/platelets ratio index')

**The list of articles excluded after full-text reading**

- **Not enough data for risk effects along with corresponding 95%CI for OS or DFS**

1. Cheng J, Zhao P, Liu J, Liu X, Wu X. Preoperative aspartate aminotransferase-to-platelet ratio index (APRI) is a predictor on postoperative outcomes of hepatocellular carcinoma. Medicine (Baltimore). 2016 Nov;95(48):e5486. doi: 10.1097/MD.0000000000005486.
2. Mai RY, Ye JZ, Long ZR, Shi XM, Bai T, Chen J, Li LQ, Wu GB, Wu FX. Preoperative aspartate aminotransferase-to-platelet-ratio index as a predictor of posthepatectomy liver failure for resectable hepatocellular carcinoma. Cancer Manag Res. 2019 Feb 12;11:1401-1414. doi: 10.2147/CMAR.S186114.
3. Angulo P, Bugianesi E, Bjornsson ES, Charatcharoenwitthaya P, Mills PR, Barrera F, Haflidadottir S, Day CP, George J. Simple noninvasive systems predict long-term outcomes of patients with nonalcoholic fatty liver disease. Gastroenterology. 2013 Oct;145(4):782-9.e4. doi: 10.1053/j.gastro.
4. Pagano AP, Sicchieri JMF, Schiavoni IL, Barbeiro D, Manca CS, da Silva BR, Bezerra AE, Pinto LCM, Araújo RC, Teixeira AC, Chiarello PG. Phase angle as a severity indicator for liver diseases. Nutrition. 2020 Feb;70:110607. doi: 10.1016/j.nut.2019.110607.
5. Tanaka S, Iimuro Y, Hirano T, Hai S, Suzumura K, Fujimoto J. Prediction of Postoperative Hepatic Failure after Liver Resection for Hepatocellular Carcinoma: Significance of the Aspartate Aminotransferase-to-Platelet Ratio Index. Hepatogastroenterology. 2014 May;61(131):755-61.
6. Mai RY, Wang YY, Bai T, Chen J, Xiang BD, Wu GB, Wu FX, Li LQ, Ye JZ. Combination Of ALBI And APRI To Predict Post-Hepatectomy Liver Failure After Liver Resection For HBV-Related HCC Patients. Cancer Manag Res. 2019 Oct 2;11:8799-8806. doi: 10.2147/CMAR.S213432.
7. Zhang ZQ, Yang B, Zou H, Xiong L, Miao XY, Wen Y, Zhou JJ. ALBI/ST ratio versus FIB-4 and APRI as a predictor of posthepatectomy liver failure in hepatocellular carcinoma patients. Medicine (Baltimore). 2019 Apr;98(15):e15168. doi: 10.1097/MD.0000000000015168.
8. Sun LY, Zhu H, Diao YK, Xing H, Liang L, Li J, Zhou YH, Gu WM, Chen TH, Zeng YY, Pawlik TM, Lau WY, Li C, Shen F, Zhang CW, Yang T. A novel online calculator based on albumin-bilirubin and aspartate transaminase-to-platelet ratio index for predicting postoperative morbidity following hepatectomy for hepatocellular carcinoma. Ann Transl Med. 2020 Dec;8(23):1591. doi: 10.21037/atm-20-1421.
9. Lin PT, Teng W, Jeng WJ, Hsieh YC, Hung CF, Huang CH, Lui KW, Chen YC, Lin CC, Lin SM, Sheen IS, Lin CY. The incidence and predictors of post transarterial chemoembolization variceal bleeding in hepatocellular carcinoma patients. J Formos Med Assoc. 2020 Feb;119(2):635-643. doi: 10.1016/j.jfma.2019.08.019.
10. Pereyra D, Rumpf B, Ammann M, Perrodin SF, Tamandl D, Haselmann C, Stift J, Brostjan C, Laengle F, Beldi G, Gruenberger T, Starlinger P. The Combination of APRI and ALBI Facilitates Preoperative Risk Stratification for Patients Undergoing Liver Surgery After Neoadjuvant Chemotherapy. Ann Surg Oncol. 2019 Mar;26(3):791-799. doi: 10.1245/s10434-018-07125-6.
11. Shi JY, Sun LY, Quan B, Xing H, Li C, Liang L, Pawlik TM, Zhou YH, Wang H, Gu WM, Chen TH, Lau WY, Shen F, Wang NY, Yang T. A novel online calculator based on noninvasive markers (ALBI and APRI) for predicting post-hepatectomy liver failure in patients with hepatocellular carcinoma. Clin Res Hepatol Gastroenterol. 2021 Jul;45(4):101534. doi: 10.1016/j.clinre.2020.09.001.
12. Sung KC, Johnston MP, Lee MY, Byrne CD. Non-invasive liver fibrosis scores are strongly associated with liver cancer mortality in general population without liver disease. Liver Int. 2020 Jun;40(6):1303-1315. doi: 10.1111/liv.14416.
13. Ke MY, Zhang M, Su Q, Wei S, Zhang J, Wang Y, Wu R, Lv Y. Gamma-glutamyl transpeptidase to platelet ratio predicts short-term outcomes in hepatocellular carcinoma patients undergoing minor liver resection. J Surg Res. 2018 Nov;231:403-410. doi: 10.1016/j.jss.2018.05.049.
14. Amptoulach S, Gross G, Sturesson C, Rissler P, Kalaitzakis E. Preoperative Aspartate Aminotransferase-to-Platelet Ratio Index Predicts Perioperative Liver-Related Complications Following Liver Resection for Colorectal Cancer Metastases. Scand J Surg. 2017 Dec;106(4):311-317. doi: 10.1177/1457496916683094.
15. Guo C, Liang H, Yuan W, Qin Y. Analysis on the value of soluble intercellular adhesion molecule-1 (sICAM-1), alpha fetoprotein (AFP), and aspartate aminotransferase/platelet ratio index (APRI) in predicting the prognostic survival of patients with primary liver cancer after radiofrequency ablation. Ann Palliat Med. 2021 Apr;10(4):4760-4767. doi: 10.21037/apm-21-749.
16. Zheng J, Seier K, Gonen M, Balachandran VP, Kingham TP, D'Angelica MI, Allen PJ, Jarnagin WR, DeMatteo RP. Utility of Serum Inflammatory Markers for Predicting Microvascular Invasion and Survival for Patients with Hepatocellular Carcinoma. Ann Surg Oncol. 2017 Nov;24(12):3706-3714. doi: 10.1245/s10434-017-6060-7.
17. Yada M, Miyazaki M, Motomura K, Masumoto A, Nakamuta M, Kohjima M, Sugimoto R, Aratake Y, Higashi N, Morizono S, Takao S, Yamashita N, Satoh T, Yamashita S, Kuniyoshi M, Kotoh K. The prognostic role of lactate dehydrogenase serum levels in patients with hepatocellular carcinoma who are treated with sorafenib: the influence of liver fibrosis. J Gastrointest Oncol. 2016 Aug;7(4):615-23. doi: 10.21037/jgo.2016.03.10.
18. Chapelle T, Op de Beeck B, Driessen A, Roeyen G, Bracke B, Hartman V, Huyghe I, Morrison S, Ysebaert D, Francque S. Estimation of the future remnant liver function is a better tool to predict post-hepatectomy liver failure than platelet-based liver scores. Eur J Surg Oncol. 2017 Dec;43(12):2277-2284. doi: 10.1016/j.ejso.2017.08.009.
19. Ichikawa T, Uenishi T, Takemura S, Oba K, Ogawa M, Kodai S, Shinkawa H, Tanaka H, Yamamoto T, Tanaka S, Yamamoto S, Hai S, Shuto T, Hirohashi K, Kubo S. A simple, noninvasively determined index predicting hepatic failure following liver resection for hepatocellular carcinoma. J Hepatobiliary Pancreat Surg. 2009;16(1):42-8. doi: 10.1007/s00534-008-0003-4.
20. Miura K, Nakano H, Sakurai J, Kobayashi S, Koizumi S, Arai T, Shimamura T, Makizumi R, Yamada K, Miyajima N, Otsubo T, Koike J. Splenomegaly in FOLFOX-naïve stage IV or recurrent colorectal cancer patients due to chemotherapy-associated hepatotoxicity can be predicted by the aspartate aminotransferase to platelet ratio before chemotherapy. Int J Clin Oncol. 2011 Jun;16(3):257-63. doi: 10.1007/s10147-010-0176-0.
21. Vogeler M, Mohr I, Pfeiffenberger J, Sprengel SD, Klauss M, Teufel A, Chang DH, Springfeld C, Longerich T, Merle U, Mehrabi A, Weiss KH, Mieth M. Applicability of scoring systems predicting outcome of transarterial chemoembolization for hepatocellular carcinoma. J Cancer Res Clin Oncol. 2020 Apr;146(4):1033-1050. doi: 10.1007/s00432-020-03135-8.
22. Kabir T, Syn NL, Tan ZZX, Tan HJ, Yen C, Koh YX, Kam JH, Teo JY, Lee SY, Cheow PC, Chow PKH, Chung AYF, Ooi LL, Chan CY, Goh BKP. Predictors of post-operative complications after surgical resection of hepatocellular carcinoma and their prognostic effects on outcome and survival: A propensity-score matched and structural equation modelling study. Eur J Surg Oncol. 2020 Sep;46(9):1756-1765. doi: 10.1016/j.ejso.2020.03.219.

-**Did not investigate effect of APRI levels or analyze APRI separately**

1. Luo H, Li C, Chen L. Preoperative albumin-bilirubin grade combined with aspartate aminotransferase-to-platelet count ratio index predict outcomes of patients with hepatocellular carcinoma within Milan criteria after liver resection. Biosci Trends. 2019 May 12;13(2):176-181. doi: 10.5582/bst.2019.01088.
2. Pang Q, Bi JB, Xu XS, Liu SS, Zhang JY, Zhou YY, Qu K, Liu C. King's score as a novel prognostic model for patients with hepatitis B-associated hepatocellular carcinoma. Eur J Gastroenterol Hepatol. 2015 Nov;27(11):1337-46. doi: 10.1097/MEG.0000000000000452.
3. Chon YE, Jung ES, Park JY, Kim DY, Ahn SH, Han KH, Chon CY, Jung KS, Kim SU. The accuracy of noninvasive methods in predicting the development of hepatocellular carcinoma and hepatic decompensation in patients with chronic hepatitis B. J Clin Gastroenterol. 2012 Jul;46(6):518-25. doi: 10.1097/MCG.0b013e31825079f1.
4. Ke MY, Wu XN, Zhang Y, Wang S, Lv Y, Dong J. Serum GP73 predicts posthepatectomy outcomes in patients with hepatocellular carcinoma. J Transl Med. 2019 May 2;17(1):140. doi: 10.1186/s12967-019-1889-0.
5. Zhang T, Liu Z, Zhao X, Mao Z, Bai L. A novel prognostic score model based on combining systemic and hepatic inflammation markers in the prognosis of HBV-associated hepatocellular carcinoma patients. Artif Cells Nanomed Biotechnol. 2019 Dec;47(1):2246-2255. doi: 10.1080/21691401.
6. Zhou L, Wang SB, Chen SG, Qu Q, Rui JA. The prognostic value and non-invasive predictors of splenomegaly in cirrhotic patients with hepatocellular carcinoma following curative resection. Adv Clin Exp Med. 2020 Jul;29(7):879-886. doi: 10.17219/acem/81935.
7. Liu L, Wang W, Zhang Y, Long J, Zhang Z, Li Q, Chen B, Li S, Hua Y, Shen S, Peng B. Declined Preoperative Aspartate Aminotransferase to Neutrophil Ratio Index Predicts Poor Prognosis in Patients with Intrahepatic Cholangiocarcinoma after Hepatectomy. Cancer Res Treat. 2018 Apr;50(2):538-550. doi: 10.4143/crt.2017.106.
8. Yu YQ, Li J, Liao Y, Chen Q, Liao WJ, Huang J. The preoperative alkaline phosphatase-to-platelet ratio index is an independent prognostic factor for hepatocellular carcinoma after hepatic resection. Medicine (Baltimore). 2016 Dec;95(51):e5734. doi: 10.1097/MD.0000000000005734.
9. Lo Re V 3rd, Kallan MJ, Tate JP, Lim JK, Goetz MB, Klein MB, Rimland D, Rodriguez-Barradas MC, Butt AA, Gibert CL, Brown ST, Park LS, Dubrow R, Reddy KR, Kostman JR, Justice AC, Localio AR. Predicting Risk of End-Stage Liver Disease in Antiretroviral-Treated Human Immunodeficiency Virus/Hepatitis C Virus-Coinfected Patients. Open Forum Infect Dis. 2015 Jul 9;2(3):ofv109. doi: 10.1093/ofid/ofv109.
10. Nishikawa H, Osaki Y, Komekado H, Sakamoto A, Saito S, Nishijima N, Nasu A, Arimoto A, Kita R, Kimura T. Clinical significance of the FIB-4 index for non-B non-C hepatocellular carcinoma treated with surgical resection. Oncol Rep. 2015 Jan;33(1):88-94. doi: 10.3892/or.2014.3573.
11. Ito T, Kumada T, Toyoda H, Tada T. FIB-4 index for assessing the prognosis of hepatocellular carcinoma in patients with Child-Pugh class A liver function. J Cancer Res Clin Oncol. 2015 Jul;141(7):1311-9. doi: 10.1007/s00432-015-1922-5.
12. Tamaki N, Kurosaki M, Matsuda S, Muraoka M, Yasui Y, Suzuki S, Hosokawa T, Ueda K, Tsuchiya K, Nakanishi H, Itakura J, Takahashi Y, Asahina Y, Izumi N. Non-invasive prediction of hepatocellular carcinoma development using serum fibrosis marker in chronic hepatitis C patients. J Gastroenterol. 2014 Nov;49(11):1495-503. doi: 10.1007/s00535-013-0914-y.
13. Guo P, Shen SL, Zhang Q, Zeng FF, Zhang WJ, Hu XM, Zhang DM, Peng BG, Hao YT. Prognostic evaluation of categorical platelet-based indices using clustering methods based on the Monte Carlo comparison for hepatocellular carcinoma. Asian Pac J Cancer Prev. 2014;15(14):5721-7. doi: 10.7314/apjcp.2014.15.14.5721.
14. Göbel T, Vorderwülbecke S, Hauck K, Fey H, Häussinger D, Erhardt A. New multi protein patterns differentiate liver fibrosis stages and hepatocellular carcinoma in chronic hepatitis C serum samples. World J Gastroenterol. 2006 Dec 21;12(47):7604-12. doi: 10.3748/wjg.v12.i47.7604.

-**Not relevant to the prognosis of HCC**

1. Ishizuka M, Kubota K, Kita J, Shimoda M, Kato M, Mori S, Iso Y, Yamagishi H, Kojima M. Aspartate aminotransferase-to-platelet ratio index is associated with liver cirrhosis in patients undergoing surgery for hepatocellular carcinoma. J Surg Res. 2015 Mar;194(1):63-8. doi: 10.1016/j.jss.2014.09.009.
2. Gawrieh S, Wilson LA, Cummings OW, Clark JM, Loomba R, Hameed B, Abdelmalek MF, Dasarathy S, Neuschwander-Tetri BA, Kowdley K, Kleiner D, Doo E, Tonascia J, Sanyal A, Chalasani N; NASH Clinical Research Network. Histologic Findings of Advanced Fibrosis and Cirrhosis in Patients With Nonalcoholic Fatty Liver Disease Who Have Normal Aminotransferase Levels. Am J Gastroenterol. 2019 Oct;114(10):1626-1635. doi: 10.14309/ajg.0000000000000388.
3. Mai RY, Zeng J, Lu HZ, Liang R, Lin Y, Piao XM, Gao X, Wu GB, Wu FX, Ma L, Xiang BD, Li LQ, Ye JZ. Combining Aspartate Aminotransferase-to-Platelet Ratio Index with Future Liver Remnant to Assess Preoperative Hepatic Functional Reserve in Patients with Hepatocellular Carcinoma. J Gastrointest Surg. 2021 Mar;25(3):688-697. doi: 10.1007/s11605-020-04575-w.
4. Ioannou GN, Green P, Kerr KF, Berry K. Models estimating risk of hepatocellular carcinoma in patients with alcohol or NAFLD-related cirrhosis for risk stratification. J Hepatol. 2019 Sep;71(3):523-533. doi: 10.1016/j.jhep.2019.05.008. Epub 2019 May 28.
5. Kim MN, Lee JH, Chon YE, Ha Y, Hwang SG. Fibrosis-4, aspartate transaminase-to-platelet ratio index, and gamma-glutamyl transpeptidase-to-platelet ratio for risk assessment of hepatocellular carcinoma in chronic hepatitis B patients: comparison with liver biopsy. Eur J Gastroenterol Hepatol. 2020 Mar;32(3):433-439. doi: 10.1097/MEG.0000000000001520.
6. Sripongpun P, Tangkijvanich P, Chotiyaputta W, Charatcharoenwitthaya P, Chaiteerakij R, Treeprasertsuk S, Bunchorntavakul C, Sobhonslidsuk A, Leerapun A, Khemnark S, Poovorawan K, Siramolpiwat S, Chirapongsathorn S, Pan-Ngum W, Soonthornworasiri N, Sukeepaisarnjaroen W; THASL study group. Evaluation of aspartate aminotransferase to platelet ratio index and fibrosis 4 scores for hepatic fibrosis assessment compared with transient elastography in chronic hepatitis C patients. JGH Open. 2019 Jun 26;4(1):69-74. doi: 10.1002/jgh3.12219.
7. Chalouni M, Sogni P, Miailhes P, Lacombe K, Poizot-Martin I, Chas J, Vittecoq D, Neau D, Aumaitre H, Alric L, Piroth L, Bouchaud O, Katlama C, Morlat P, Lascoux-Combe C, Gervais A, Naqvi A, Rosenthal E, Garipuy D, Barange K, Esterle L, Salmon D, Wittkop L; ANRS CO13 HEPAVIH study group. Liver stiffness and fibrosis-4 alone better predict liver events compared with aspartate aminotransferase to platelet ratio index in a cohort of human immunodeficiency virus and hepatitis C virus co-infected patients from ANRS CO13 HEPAVIH cohort. Eur J Gastroenterol Hepatol. 2019 Nov;31(11):1387-1396. doi: 10.1097/MEG.0000000000001408.
8. Lemoine M, Assoumou L, De Wit S, Girard PM, Valantin MA, Katlama C, Necsoi C, Campa P, Huefner AD, Schulze Zur Wiesch J, Rougier H, Bastard JP, Stocker H, Mauss S, Serfaty L, Ratziu V, Menu Y, Schlue J, Behrens G, Bedossa P, Capeau J, Ingiliz P, Costagliola D; ANRS-ECHAM Group. Diagnostic Accuracy of Noninvasive Markers of Steatosis, NASH, and Liver Fibrosis in HIV-Monoinfected Individuals at Risk of Nonalcoholic Fatty Liver Disease (NAFLD): Results From the ECHAM Study. J Acquir Immune Defic Syndr. 2019 Apr 1;80(4):e86-e94. doi: 10.1097/QAI.0000000000001936.
9. Yoshimasu Y, Furuichi Y, Kasai Y, Takeuchi H, Sugimoto K, Nakamura I, Itoi T. Predictive factors for hepatocellular carcinoma occurrence or recurrence after direct-acting antiviral agents in patients with chronic hepatitis C. J Gastrointestin Liver Dis. 2019 Mar;28(1):63-71. doi: 10.15403/jgld.2014.1121.281.hpc.
10. Ng KJ, Tseng CW, Chang TT, Tzeng SJ, Hsieh YH, Hung TH, Huang HT, Wu SF, Tseng KC. Aspartate aminotransferase to platelet ratio index and sustained virologic response are associated with progression from hepatitis C associated liver cirrhosis to hepatocellular carcinoma after treatment with pegylated interferon plus ribavirin. Clin Interv Aging. 2016 Aug 1;11:1035-41. doi: 10.2147/CIA.S108589.
11. Rao H, Xie Q, Shang J, Gao Z, Chen H, Sun Y, Jiang J, Niu J, Zhang L, Wang L, Zhao L, Li J, Yang R, Zhu S, Li R, Wei L. Real-world clinical outcomes among individuals with chronic HCV infection in China: CCgenos study. Antivir Ther. 2019;24(7):473-483. doi: 10.3851/IMP3334.
12. Nartey YA, Awuku YA, Agyei-Nkansah A, Duah A, Bampoh SA, Ayawin J, Asibey SO, Björkström NK, Ye W, Afihene MY, Roberts LR, Plymoth A. Ambulatory end-stage liver disease in Ghana; patient profile and utility of alpha fetoprotein and aspartate aminotransferase: platelet ratio index. BMC Gastroenterol. 2020 Dec 26;20(1):428. doi: 10.1186/s12876-020-01581-9.
13. Ji F, Zhou R, Wang W, Bai D, He C, Cai Z, Shen Y, Wang S, Deng H, Li Z. High Post-treatment α-Fetoprotein Levels and Aspartate Aminotransferase-to-Platelet Ratio Index Predict Hepatocellular Carcinoma in Hepatitis C Virus Decompensated Cirrhotic Patients with Sustained Virological Response After Antiviral Therapy. J Interferon Cytokine Res. 2017 Aug;37(8):362-368. doi: 10.1089/jir.2017.0040.
14. Li X, Xu H, Gao P. Fibrosis Index Based on 4 Factors (FIB-4) Predicts Liver Cirrhosis and Hepatocellular Carcinoma in Chronic Hepatitis C Virus (HCV) Patients. Med Sci Monit. 2019 Sep 27;25:7243-7250. doi: 10.12659/MSM.918784.
15. Nishikawa H, Nishijima N, Enomoto H, Sakamoto A, Nasu A, Komekado H, Nishimura T, Kita R, Kimura T, Iijima H, Nishiguchi S, Osaki Y. Comparison of FIB-4 index and aspartate aminotransferase to platelet ratio index on carcinogenesis in chronic hepatitis B treated with entecavir. J Cancer. 2017 Jan 11;8(2):152-161. doi: 10.7150/jca.16523.
16. Chun HS, Kim BK, Park JY, Kim DY, Ahn SH, Han KH, Lee CH, Lee YB, Cho EJ, Yu SJ, Kim YJ, Yoon JH, Lee JH, Kim SU. Design and validation of risk prediction model for hepatocellular carcinoma development after sustained virological response in patients with chronic hepatitis C. Eur J Gastroenterol Hepatol. 2020 Mar;32(3):378-385. doi: 10.1097/MEG.0000000000001512.
17. Huang TH, Lin MT, Wang JH, Chang KC, Yen YH, Kuo FY, Huang CC, Hsiao CC, Chiu SY, Lu SN, Wang CC, Hu TH. Clinical and novel application of FibroScan, FIB-4 and aspartate aminotransferase-to-platelet ratio index in liver fibrosis evaluation in patients with hepatocellular carcinoma and their roles in oesophageal variceal prediction. Int J Clin Pract. 2021 Apr;75(4):e13945. doi: 10.1111/ijcp.13945.
18. Cheung KS, Seto WK, Fung J, Mak LY, Lai CL, Yuen MF. Prediction of hepatocellular carcinoma development by aminotransferase to platelet ratio index in primary biliary cholangitis. World J Gastroenterol. 2017 Nov 28;23(44):7863-7874. doi: 10.3748/wjg.v23.i44.7863.
19. Hann HW, Wan S, Lai Y, Hann RS, Myers RE, Patel F, Zhang K, Ye Z, Wang C, Yang H. Aspartate aminotransferase to platelet ratio index as a prospective predictor of hepatocellular carcinoma risk in patients with chronic hepatitis B virus infection. J Gastroenterol Hepatol. 2015 Jan;30(1):131-138. doi: 10.1111/jgh.12664.
20. Unalp-Arida A, Ruhl CE. Liver fibrosis scores predict liver disease mortality in the United States population. Hepatology. 2017 Jul;66(1):84-95. doi: 10.1002/hep.
21. Park YE, Kim BK, Park JY, Kim DY, Ahn SH, Han KH, Han S, Jeon MY, Heo JY, Song K, Kim SU. Gamma-glutamyl transpeptidase-to-platelet ratio is an independent predictor of hepatitis B virus-related liver cancer. J Gastroenterol Hepatol. 2017 Jun;32(6):1221-1229. doi: 10.1111/jgh.13653.
22. Lee JJ, Wei YJ, Lin MY, Niu SW, Hsu PY, Huang JC, Jang TY, Yeh ML, Huang CI, Liang PC, Lin YH, Hsieh MY, Hsieh MH, Chen SC, Dai CY, Lin ZY, Chen SC, Huang JF, Chang JM, Hwang SJ, Huang CF, Chiu YW, Chuang WL, Yu ML. The applicability of non-invasive methods for assessing liver fibrosis in hemodialysis patients with chronic hepatitis C. PLoS One. 2020 Nov 20;15(11):e0242601. doi: 10.1371/journal.pone.0242601.
23. Wu CK, Chang KC, Hung CH, Tseng PL, Lu SN, Chen CH, Wang JH, Lee CM, Tsai MC, Lin MT, Yen YH, Hu TH. Dynamic α-fetoprotein, platelets and AST-to-platelet ratio index predict hepatocellular carcinoma in chronic hepatitis C patients with sustained virological response after antiviral therapy. J Antimicrob Chemother. 2016 Jul;71(7):1943-7. doi: 10.1093/jac/dkw097.
24. Lee K, Sinn DH, Gwak GY, Cho HC, Jung SH, Paik YH, Choi MS, Lee JH, Koh KC, Paik SW. Prediction of the Risk of Hepatocellular Carcinoma in Chronic Hepatitis C Patients after Sustained Virological Response by Aspartate Aminotransferase to Platelet Ratio Index. Gut Liver. 2016 Sep 15;10(5):796-802. doi: 10.5009/gnl15368.
25. Mobarak L, Omran D, Nabeel MM, Zakaria Z. Fibro markers for prediction of hepatocellular carcinoma in Egyptian patients with chronic liver disease. J Med Virol. 2017 Jun;89(6):1062-1068. doi: 10.1002/jmv.24720.
26. Gara N, Zhao X, Kleiner DE, Liang TJ, Hoofnagle JH, Ghany MG. Discordance among transient elastography, aspartate aminotransferase to platelet ratio index, and histologic assessments of liver fibrosis in patients with chronic hepatitis C. Clin Gastroenterol Hepatol. 2013 Mar;11(3):303-8.e1. doi: 10.1016/j.cgh.2012.10.044.
27. Toyoda H, Tada T, Tachi Y, Hirai T, Yasuda S, Honda T, Hayashi K, Ishigami M, Goto H, Kumada T. Liver fibrosis indices for identifying patients at low risk of developing hepatocellular carcinoma after eradication of HCV. Antivir Ther. 2017;22(3):185-193. doi: 10.3851/IMP3081.
28. Abdelgawad IA. Clinical utility of simple non-invasive liver fibrosis indices for predicting hepatocellular carcinoma (HCC) among Egyptian patients. J Clin Pathol. 2015 Feb;68(2):154-60. doi: 10.1136/jclinpath-2014-202462.
29. Kim M, Lee Y, Yoon JS, Lee M, Kye SS, Kim SW, Cho Y. The FIB-4 Index Is a Useful Predictor for the Development of Hepatocellular Carcinoma in Patients with Coexisting Nonalcoholic Fatty Liver Disease and Chronic Hepatitis B. Cancers (Basel). 2021 May 11;13(10):2301. doi: 10.3390/cancers13102301.
30. Sato M, Hikita H, Hagiwara S, Sato M, Soroida Y, Suzuki A, Gotoh H, Iwai T, Kojima S, Matsuura T, Yotsuyanagi H, Koike K, Yatomi Y, Ikeda H. Potential associations between perihepatic lymph node enlargement and liver fibrosis, hepatocellular injury or hepatocarcinogenesis in chronic hepatitis B virus infection. Hepatol Res. 2015 Apr;45(4):397-404. doi: 10.1111/hepr.12361.
31. Lin CS, Chang CS, Yang SS, Yeh HZ, Lin CW. Retrospective evaluation of serum markers APRI and AST/ALT for assessing liver fibrosis and cirrhosis in chronic hepatitis B and C patients with hepatocellular carcinoma. Intern Med. 2008;47(7):569-75. doi: 10.2169/internalmedicine.47.0595.
32. de Lédinghen V, Vergniol J, Barthe C, Foucher J, Chermak F, Le Bail B, Merrouche W, Bernard PH. Non-invasive tests for fibrosis and liver stiffness predict 5-year survival of patients chronically infected with hepatitis B virus. Aliment Pharmacol Ther. 2013 May;37(10):979-88. doi: 10.1111/apt.12307.
33. Han WM, Ueaphongsukkit T, Chattranukulchai P, Siwamogsatham S, Chaiteerakij R, Sophonphan J, Gatechompol S, Ubolyam S, Phonphithak S, Ruxrungtham K, Tangkijvanich P, Avihingsanon Y, Kerr SJ, Avihingsanon A. Incident Liver Cirrhosis, Associated Factors, and Cardiovascular Disease Risks Among People Living With HIV: A Longitudinal Study. J Acquir Immune Defic Syndr. 2021 Apr 1;86(4):463-472. doi: 10.1097/QAI.0000000000002585.
34. Kim JH, Kim JW, Seo JW, Choe WH, Kwon SY. Noninvasive Tests for Fibrosis Predict 5-Year Mortality and Hepatocellular Carcinoma in Patients With Chronic Hepatitis B. J Clin Gastroenterol. 2016 Nov/Dec;50(10):882-888. doi: 10.1097/MCG.0000000000000574.
35. Tada T, Kumada T, Toyoda H, Kiriyama S, Tanikawa M, Hisanaga Y, Kanamori A, Kitabatake S, Yama T, Tanaka J. Viral eradication reduces all-cause mortality, including non-liver-related disease, in patients with progressive hepatitis C virus-related fibrosis. J Gastroenterol Hepatol. 2017 Mar;32(3):687-694. doi: 10.1111/jgh.13589.
36. Yamada S, Kawaguchi A, Kawaguchi T, Fukushima N, Kuromatsu R, Sumie S, Takata A, Nakano M, Satani M, Tonan T, Fujimoto K, Shima H, Kakuma T, Torimura T, Charlton MR, Sata M. Serum albumin level is a notable profiling factor for non-B, non-C hepatitis virus-related hepatocellular carcinoma: A data-mining analysis. Hepatol Res. 2014 Aug;44(8):837-45. doi: 10.1111/hepr.12192.
37. Nam H, Lee SW, Kwon JH, Lee HL, Yoo SH, Kim HY, Song DS, Sung PS, Chang UI, Kim CW, Nam SW, Bae SH, Choi JY, Yoon SK, Yang JM, Han NI, Jang JW. Prediction of Hepatocellular Carcinoma by On-Therapy Response of Noninvasive Fibrosis Markers in Chronic Hepatitis B. Am J Gastroenterol. 2021 Mar 10. doi: 10.14309/ajg.0000000000001219.
38. Soubrane O, Brouquet A, Zalinski S, Terris B, Brézault C, Mallet V, Goldwasser F, Scatton O. Predicting high grade lesions of sinusoidal obstruction syndrome related to oxaliplatin-based chemotherapy for colorectal liver metastases: correlation with post-hepatectomy outcome. Ann Surg. 2010 Mar;251(3):454-60. doi: 10.1097/SLA.0b013e3181c79403.
39. Jeong J, Shin JW, Jung SW, Lee SB, Park EJ, Park NH. Clinical Usefulness of Non-invasive Fibrosis Indices for Predicting Hepatocellular Carcinoma in Treatment-naïve Patients with Chronic Hepatitis B Following Entecavir Therapy. Hepatol Res. 2021 Jul 5. doi: 10.1111/hepr.13690.
40. Na SK, Lee SJ, Cho YK, Kim YN, Choi EK, Song BC. Aspartate Aminotransferase-to-Platelet Ratio or Fibros-4 Index Predicts the Development of Hepatocellular Carcinoma in Chronic Hepatitis C Patients with Sustained Virologic Response to Interferon Therapy. J Interferon Cytokine Res. 2019 Nov;39(11):703-710. doi: 10.1089/jir.2019.0049.

-**With overlapping patients**

1. Ji F, Fu S, Guo Z, Pang H, Chen D, Wang X, Ju W, Wang D, He X, Hua Y, Peng B. Prognostic significance of preoperative aspartate aminotransferase to neutrophil ratio index in patients with hepatocellular carcinoma after hepatic resection. Oncotarget. 2016 Nov 1;7(44):72276-72289. doi: 10.18632/oncotarget.10848.
2. Pang Q, Zhang JY, Xu XS, Song SD, Chen W, Zhou YY, Miao RC, Qu K, Liu SS, Dong YF, Liu C. The prognostic values of 12 cirrhosis-relative noninvasive models in patients with hepatocellular carcinoma. Scand J Clin Lab Invest. 2015 Jan;75(1):73-84. doi: 10.3109/00365513.2014.
